# Mapping Chemical Selection Pathways for Designing Multicomponent Alloys: an informatics framework for materials design

**DOI:** 10.1038/srep17960

**Published:** 2015-12-18

**Authors:** Srikant Srinivasan, Scott R. Broderick, Ruifeng Zhang, Amrita Mishra, Susan B. Sinnott, Surendra K. Saxena, James M. LeBeau, Krishna Rajan

**Affiliations:** 1 Plant Sciences Institute, Iowa State University, 2031 Roy J. Carver Co-Lab, Ames, IA 50011; 2 Department of Materials Design and Innovation, University at Buffalo- State University of New York, 311 Bell Hall, Buffalo, NY 14260; 3 School of Materials Science and Engineering, Beihang University, Beijing 100191, People’s Republic of China; 4 Department of Mechanical Engineering, University of Mississippi, 201C Carrier, University, MS 38677; 5 Department of Materials Science and Engineering, Pennsylvania State University, 111 Research Unit A, University Park, PA 16801; 6 Department of Mechanical and Materials Engineering, Florida International University, 140 Building VH, Miami, FL 33199; 7 Department of Materials Science and Engineering, North Carolina State University, 3076A EB 1, Raleigh, NC 27606.

## Abstract

A data driven methodology is developed for tracking the collective influence of the multiple attributes of alloying elements on both thermodynamic and mechanical properties of metal alloys. Cobalt-based superalloys are used as a template to demonstrate the approach. By mapping the high dimensional nature of the systematics of elemental data embedded in the periodic table into the form of a network graph, one can guide targeted first principles calculations that identify the influence of specific elements on phase stability, crystal structure and elastic properties. This provides a fundamentally new means to rapidly identify new stable alloy chemistries with enhanced high temperature properties. The resulting visualization scheme exhibits the grouping and proximity of elements based on their impact on the properties of intermetallic alloys. Unlike the periodic table however, the distance between neighboring elements uncovers relationships in a complex high dimensional information space that would not have been easily seen otherwise. The predictions of the methodology are found to be consistent with reported experimental and theoretical studies. The informatics based methodology presented in this study can be generalized to a framework for data analysis and knowledge discovery that can be applied to many material systems and recreated for different design objectives.

The search for elemental substitutions and/or additions needed to refine metal alloy compositions and enhance their properties is a classical problem in metallurgical alloy design. Finding appropriate alloy chemistries based on a systematic exploration using either computational and/or experimental approaches is often guided by prior heuristic knowledge that harnesses expected trends captured in the periodic table that can influence phase stability and properties. Despite decades of work we have, as of yet, no unified mathematical formalism for harnessing this heuristic knowledge and thus more rapidly target our next potential discovery of an alloy. Our work identifies possible compositions for intermetallic formation. We employ manifold learning methods as a screening procedure for where detailed first principles calculations need to be focused, rather than run thousands of calculations of numerous permutations of compositions and then apply machine learning algorithms to search for potential minimum energy structures. In this paper we lay out this methodology for addressing the Grand Challenge of accelerating alloy design.

The recent discovery by Sato *et al.*^1^ of the existence of a Co_3_(Al,W) L1_2_ intermetallic has spawned a renewed interest in cobalt based superalloys for high temperature applications after many decades of relative dormancy^2^. It serves as a good example of how challenging multicomponent alloy design can be. Sato *et al.* found that with the addition of W, Co_3_(Al,W) is indeed a stable intermetallic possessing all the characteristics needed (e.g. high melting point, L1_2_ ordered structure, appropriate lattice parameter to achieve coherency strains) to enhance high temperature mechanical properties of cobalt alloys typical to nickel based superalloys. The determination that W was the key element required a patient and detailed experimental search. It was not obvious from simple inspection of known data or from the examination of property trends of elements from the periodic table, despite the decades of theoretical and empirical research in the field of alloy optimization and design. The exciting findings of Sato *et al.* serves to highlight the broader challenge in alloy design, namely how to identify the correct combination of alloying elements on intermetallic chemistry that governs both phase stability and such critical factors as mechanical and physical properties. No existing theoretical framework is able to simultaneously capture all of these multidimensional metrics of thermodynamics, crystal structure and microstructure.

The approach described here is designed to meet this Grand Challenge. In particular, we build on our extensive prior work applying statistical learning methods to critically assess and rank the influence of numerous and diverse parameters ranging from crystal chemistry to electronic structure descriptors on their potential influence on the multi-objective property targets of thermodynamic stability and physical and mechanical properties of intermetallics. We identify here potential alloying additions and thus target the chemistries for which thermodynamic calculations need to be done while significantly shrinking the chemical search space. One of the major benefits of our work is that the directed graph representation employed here readily scales with both binary and multicomponent pseudo-binary phase diagrams, and most importantly, identifies chemical phase spaces that have a likelihood of having intermetallics that meet the requirements for enhanced high temperature mechanical properties.

## Data Description and Methods

**Figure 1:**
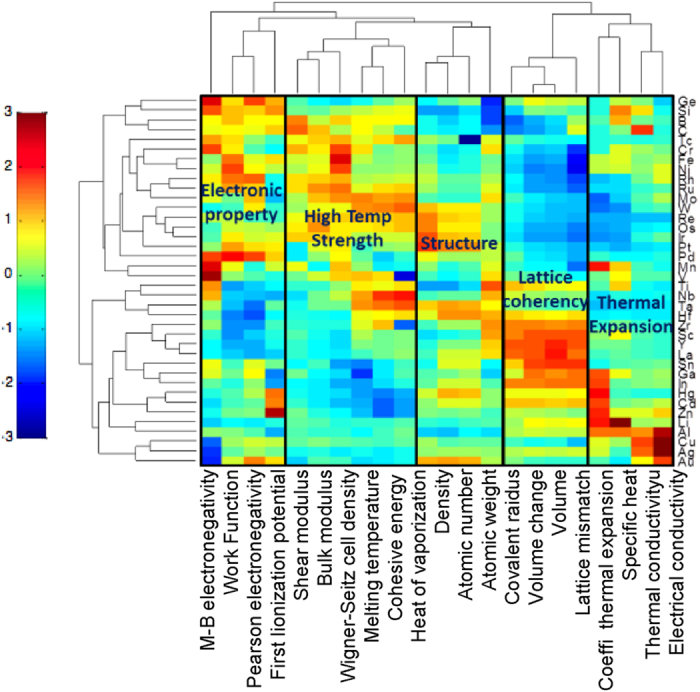
A heat map derived from the correlation matrix associated with the high dimensional input data, combining descriptors such as from Villars, Mooser-Pearson, Pettifor, Hume-Rothery and Miedema^3^,^4^,^5^,^6^,^7^,^8^,^9^. The ordering of the descriptors and the elements is based on their similarities, as described by the dendrograms. The heat map shows 22 properties for 38 elements/compounds. The descriptor set covers the property categories of electronic, high temperature strength, structure, lattice coherency and thermal expansion. To ensure that no particular properties are overweighting our analysis, the values are mean centered and standardized. For this reason, the properties all fall within a comparable range, as shown in the color scale. This step ensures robustness and enables interrogation of the design pathways.

The selection of data (or “descriptors”) was organized into three broad classes of information: discrete scalar parameters that relate to solid state properties of single elements, thermodynamic and physical properties of potential alloy chemistries using Miedema’s^3^,^4^ model coupled to alloy design rules from the classical theories on phase stability of Villars^5^, Mooser-Pearson^6^,^7^, Pettifor^8^, and Hume-Rothery^9^, and finally verification with a dimensionless descriptor database that captures the electronic structure via eigenvalue decomposition of spectral features from density of states curves of a small training set of both individual elements and of a few binary intermetallic alloys. For example, Fig. 1 illustrates a heat map of pairwise correlations of the influence of alloying elements (X) in Co_3_(Al,X) and the properties represented by dendrograms which categorize the input data into the different genres playing a significant role in alloying characteristics.

The interpretation of this heat map can best be understood if one recognizes that each alloying element ‘*i*’ forming a row of the database is associated with a set of properties. Each of these properties or descriptors, forming a column of the heat map, can be represented by an axis of a high dimensional Euclidean space *R*^*n*^, where ‘*n’* is the total number of descriptors. Correspondingly each element ‘*i*’ can be represented by a data point *x*_*i*_ mapped out in this high dimensional descriptor space *R*^*n*^ where the coordinates of *x*_*i*_ are given by the magnitudes of the various descriptors in relation to element ‘*i*’. The challenge is that one heat map of one class of descriptors alone does not capture the curvature of the hyper plane on which the data sits and the similarity metrics need to be captured by geodesic distances. Hence there is the need to apply non-linear manifold projection methods.

Using these criteria as the basis for mapping similarity among the alloying elements, we screened for trajectories of interest, such as high cohesive energy, by interrogating a dissimilarity graph generated through manifold learning methods. In our prior work we have explored numerous methods to explore ways to ascertain how to statistically assess the interaction of such multivariate data, including dimensionality reduction mapping^10^,^11^,^12^,^13^,^14^, information entropy-based recursive partitioning^15^,^16^, and evolutionary methods^17^,^18^. In the present work we build on this foundation by applying non-linear manifold learning methods. Specifically, we use the Isomap algorithm^19^ that goes beyond the assumption that a low dimensional manifold exists and generates a low dimensional embedding of data points that preserves the best possible geodesic distance between all pairs of data points. The collection of various elemental and Co alloying descriptors form the axes of a high dimensional Euclidean space *R*^*n*^ which are mapped out in this high dimensional space as a finite set of data points *{x*_*i*_*} ϵ R*^*n*^. The relevant descriptors represent various physical properties, crystal structure and chemistry. Given only the data points *{x*_*i*_*}* and the corresponding descriptors as the input, Isomap^20^,^21^ attempts to recover a smooth nonlinear submanifold *M*^*d*^ of lower dimension *d < n*, upon which the points *x*_*i*_ *ϵ R*^*n*^ lie and then unfolds *M*^*d*^ to visually capture relationships between the datapoints, while preserving the geodesic metric distances between them along the submanifold. The algorithm applies non-linear dimensionality reduction to map the set of points *{x*_*i*_*} ϵ R*^*n*^ to *{y*_*i*_*} ϵ M*^*d*^ specified by *x*_*i*_ *→ y*_*i*_ *| y*_*i*_ *ϵ M*^*d*^*, d<n, s.t.* 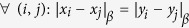 where 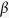 is a norm, representative of the pairwise geodesic distances 
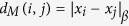
 between any two elements 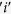 and 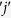 in *R*^*n*^ along the submanifold *M*^*d*^. This is performed by first constructing a weighted graph in *R*^*n*^ that connects the data points *{x*_*i*_*}* utilizing some form of nearest neighbor connectivity. The crucial stage of the Isomap algorithm is to construct the appropriate graph so that the pairwise geodesic distance between the elements along the graph, 
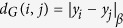
, is an accurate approximation of 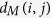. The output of Isomap algorithm is then the points *{y*_*i*_*}* plotted out on the dimensionally reduced weighted graph.

The geodesic distance is defined as the shortest distance between a pair of points along a manifold and in this case, the nonlinear manifold in the high dimensional space is obtained by connecting each element to its ‘*k*’ nearest neighbors in terms of their collective impact within the high dimensional data space associated with thermodynamic, structural and mechanical alloying properties. The algorithm aims to produce low dimensional projections of data that geometrically map the true correlations between elements in the original manifold and the resultant projection of data is shown to uncover the relative impact of elements in their role as alloying additions to Co_3_(Al,X) both in terms of phase stability and mechanical properties in a fundamentally novel manner that is not apparent from an examination of the traditional periodic table alone.

## Results and Discussion

**Figure 2:**
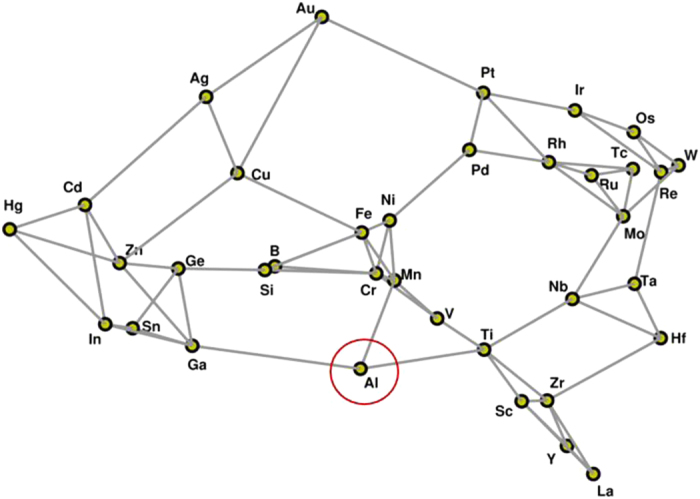
A graphing approach to capture similarity/dissimilarity metrics for alloy design. The design pathways are chosen based on expected strength and stability. This map is adaptable to finding different substitutional pathways for different design requirements as shown in Fig. 3.

The Isomap algorithm was used to discover the optimal low dimensional graph embedding of elements in their role as alloying additions to Co_3_(Al,X), such that the geodesic distance between the elements in the higher dimensional manifold is preserved when it is mapped onto the lower dimensional graph (details of the algorithmic implementation are described in the supplementary section). Each alloying element (X), for the alloy Co_3_(Al,X) becomes a graph vertex and each vertex is connected to its neighboring vertices through edges whose weights are proportional to the distance between the vertices (Fig. 2). This permits one to readily identify pathways of similarity (or dissimilarity) between elements that may serve to stabilize the L1_2_ structure for a Co_3_(Al,X) stoichiometry, which leads to identifying intermetallic chemistries that have a high cohesive energy, high melting point and a lattice parameter that will ensure coherency strains in a Co rich fcc matrix.

The uncertainty of the connections identified can be assessed by changing the number of nearest neighbor connections, as well as the number of dimensions included in the analysis. The change of connections and neighboring lengths is correlated to the uncertainty in the results. The optimal number of dimensions in which to represent the graph output of Isomap can be determined by a Scree plot which is an ordered representation of the impact of each additional dimension, in the low dimensional representation, in accurately representing the geodesic distance along the original manifold (see supplementary material). Since the manifold in high dimensional space can vary depending on the number of nearest neighbors chosen, a measure of statistical uncertainty in the geodesic distances can be obtained by varying the number of nearest neighbors to check for short-circuit errors^22^ as well as by ensuring the optimum number of dimensions for low dimensional representation. We find that the first two dimensions are sufficient to represent 90% of the original geodesic distances in all cases of nearest neighbors while the embeddings themselves show that the overall structure of the manifold does not change by varying the number of neighbors other than to increase the number of pathways. For the case of *k* = 2, the manifold becomes disconnected. Therefore, in this case we choose *k* = 3 to ensure that the resulting graph embedding is neither over-connected, leading to loss of pairwise geodesic distances, nor are critical neighbors disconnected^23^. Further, the comparison of connections under the different input parameters do not change significantly, demonstrating that the results presented here have low levels of uncertainty for every node.

Figure 2 is a network graph that shows the relative similarity/dissimilarity between elements (nodes) as potential alloying elements (X) in terms of their collective impact on the properties of Co_3_(Al,X). It should be noted that this diagram is also applicable to higher order multicomponent systems by suggesting additional elements (Y) for Co_3_(Al,X,Y) by considering both first and second nearest neighbors at each node. The key feature which we utilize in this graph is the relative distances of the connecting edges. The length of the edge represents the dissimilarity between the vertices it connects and the elements closest to each other are most similar in terms of the descriptors that go into the construction of this graph. The edges of the graph connect elements that have the strongest similarity with respect to each other. Each node identifies a ternary alloy composition of the type Co_3_(Al,X). The edges connecting two nodes Co_3_(Al,X) and Co_3_(Al,Y) for instance would be associated with a range of compositions and phases that are mapped onto a quaternary phase diagram of Co, Al, X and Y, where X and Y are the chemical additions. Hence another unique feature is that it identifies new multicomponent systems that may in fact have stable intermetallics with the desired properties we seek. This provides the framework for targeted phase diagram computations.

**Figure 3:**
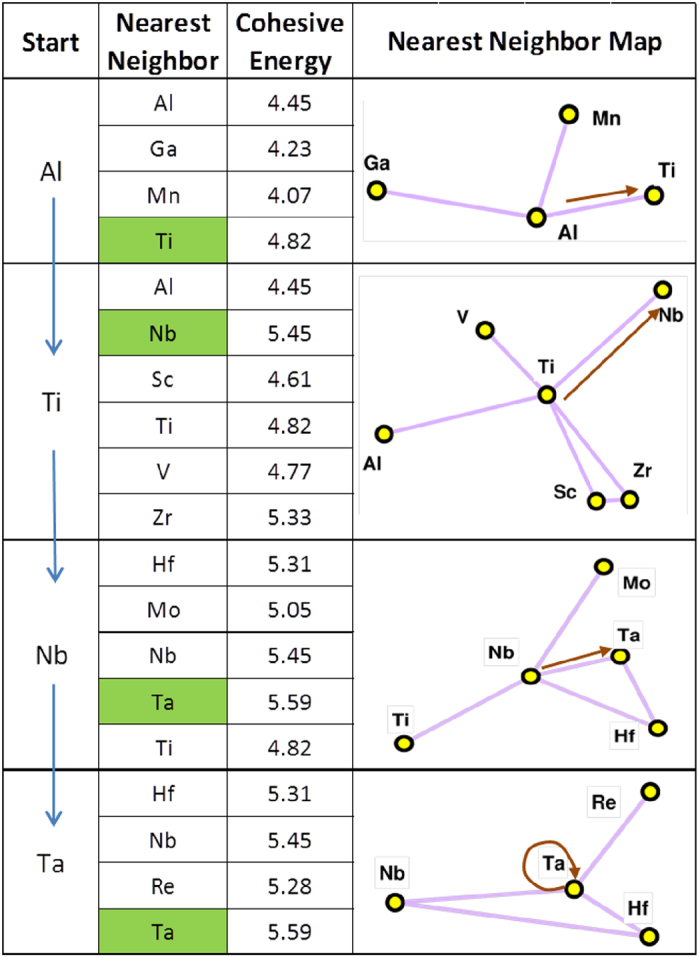
The determination of the pathway shown for relative cohesive energy going from Al to Ta in Fig. 2. The enthalpy and cohesive energies shown in this figure were calculated using Miedema’s model. Starting with Co_3_Al, we find that the cohesive energy increases most with substitution of Ti (highest cohesive energy of any of the Isomap neighbor compounds of Al). This finding agrees with our DFT calculations which show that out of eight different structures we calculated, Co_3_Al has tetragonal ground state structure, while Co_3_Ti has L1_2_ ground state structure. Following our criteria for increasing cohesive energy, we identify the pathway as going from Ti to Nb and Nb to Ta, with cohesive energy for Ta having the highest value of any compound. This figure shows how similar substitutional pathways can be defined for designing to maximize any given property.

**Figure 4:**
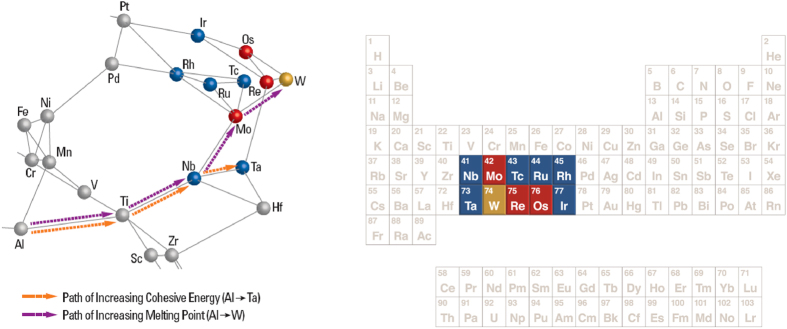
Comparison of (left) manifold representation of relative relationships of alloying elements with respect to equivalent positions as shown in the periodic table (right). The pathway for exploring other elements is not easily discernible looking at traditional systematics of the periodic table (for example rows, groups, Mendeleev number). The color coding in the figure serves to highlight the comparison with W addition, which has been shown to result in stable Co_3_Al^1^. Therefore, W is shown in gold in both the graph and periodic table, while first nearest neighbors to W are shown in red, and second nearest neighbors to W are shown in blue.

As a first step, with the objective of defining a substitute X for Co_3_(Al,X), the graph network identifies the first nearest neighbors of Al (Ga, Mn and Ti) that are most similar to Al and the dissimilarity strengthens as we move to second, third, and further nearest neighbors. In this case, we know that Co_3_Al as a L1_2_ structure is not stable, hence if we want to find other alloying elements to add, we need to probe the neighborhood of Al. The following rules are used to navigate the graph network. Since Al has multiple edges connecting to neighbors, in order to identify which direction we move in, we select the element that has a higher level of stability (from Miedema’s model), and therefore Ti serves as the first step. At the Ti node, we again identify the possible branches but also add on other levels of constraint such as modulus and cohesive energy in making the decisions for the next step (Fig. 3). Using this logic repeatedly at each node, we finally reach W, as was empirically discovered by Sato^1^. If we define our criteria as optimizing cohesive energy, we obtain an alternate pathway to W as illustrated in Fig. 4 Each intermediate node along the pathway has been suggested as a potential alloying element for Co_3_(Al,X)^24^ to increase the solvus temperature. If we define our criteria as optimizing cohesive energy, we obtain a diverging pathway leading to Ta as illustrated in Fig. 4. It is important to note that the termination of the pathway does not necessarily lead to an element representing the global maximum (or minimum) of a desired property within the graph. An element that may present the global maximum may potentially be unsuitable for alloying. The issue is not solely moving far away from the element we desire to substitute, in this case Al, as the farther we move the more difficult it is to find a similar element in terms of overall alloying properties. The aim is to track all potential elements that might provide enhanced high temperature properties while remaining as similar to Al as possible in order to provide the L1_2_ phase.

Thus the graph provides a unique map for *which* direction to move in chemical space for a specific design problem, something that a cursory inspection of the periodic table will not provide as the geometrical proximity of elements in the projection of data as visualized in the periodic table captures only the systematics of electronic structure data associated with single elements, not their collective influence on structure and properties of targeted alloy structures.

It should be added that another unique aspect of our methodology is that the network graph helps to target our thermodynamic and electronic structure computations on specific chemistries. In this approach, we are using informatics to guide and learn from the data where physical computations are needed to make decisions without having to repeat a vast number of computations over large chemical spaces. While the network graph can be interrogated to obtain pathways that may be avoided (e.g. the pathway of decreasing cohesive energy shows Mn, which is known not to strengthen the L1_2_ phase^25^), the purpose of this network is to identify chemical additions which are most likely to improve stability and high temperature properties for Co_3_Al. The objective is not to define which additives will not work. Therefore, we are reporting only those compounds which are most likely to have the best properties, while not excluding the possibility of other stable Co compounds from existing. For instance, examples have been reported where addition of Ga^26^ or Ge^27^ increase the stability of γ‘ although they are not connected to the pathway. For screening elements of interest through electronic structure calculations, the values for enthalpy of formation and cohesive energy were calculated via density functional theory (DFT). To serve as a rapid screening process, we performed calculations of binary Co_3_X, imposing an L1_2_ structure as a first approximation to Co_3_(Al,X) where additive concentration is small, in order to identify probable options just as a means of quickly assessing possible likelihoods for pathways. Following the cohesive energy pathway, we arrive at Ta, after which any additional steps lower the cohesive energy. While the nodes of the pathway are the substitutes with highest likelihood of success, the elements connected by the branches also represent potentially promising additions.

Additional information beyond confirming the stability of Co_3_(Al,W) is uncovered by identifying the pathways for different criteria, such as cohesive energy, melting temperature or other design requirements. Our work identifies possible compositions for intermetallic formation. The nodes of our graph identify potential alloying additions and thus target the chemistries for which thermodynamic calculations need to be done to confirm whether these compounds do indeed exist. Hence the manifold learning methods serve as a screening procedure for where detailed first principles calculations need to be focused, rather than run thousands of calculations of numerous permutations of compositions and then apply machine learning algorithms to search for potential minimum energy structures. Further, while we find W to be a suitable addition, we find additional nodes that look to be as promising, such as Ta and Re. However, a single design requirement is not sufficient for identifying additives, thereby requiring multiple design pathways. For example, we have shown different pathways leading to W or to Ta, depending on the design requirement. Therefore, this identifies that a combination of these additives leads to a good combination of cohesive energy (or the highly correlated melting temperature) and modulus. This demonstrates the application of the graph network for multi-functional design.

This analysis (1) confirmed Sato’s^1^ empirical studies on W addition to Co_3_Al; (2) identified different pathways for property improvement; and (3) determined chemical substitutes for Co-based superalloys. Our results are consistent with reported experimental and theoretical studies, as indicated in Table 1. The agreement of these prior studies with the graphical network result provides the foundation for application of this approach.

Shown in Fig. 4 are additional possible substitutes for quaternary systems (i.e. Co_3_(Al,X,Y)). For instance, Ta addition to quaternary Co_3_(Al,W,Y) has indeed been experimentally reported^28^. We identify the new quaternary systems by including the additives which are nearest neighbors. These are further the most suitable additions to Co_3_Al. This therefore guides the next series of experiments. In addition to the experiments suggested from our ternary pathways (for example, comparing the stability and melting temperature of Co_3_(Al,W) with Co_3_(Al,Ta)), the melting temperature and stability should be experimentally measured.

The likelihood of these compositions of intermetallics having long range order is based on the nature of similarity as characterized through manifold learning metrics. We have shown that independent studies via first principles methods that empirically explored numerous compositions do indeed match our results via informatics methods, lending support to our approach. The issue of exploring the potential role of site preference is one of the next steps in our work. However our study provides the target chemistries where such studies need to be focused.

## Conclusions

This work has shown that the use of manifold learning methods can provide a powerful means of exploring the similarity and dissimilarity of the influence of alloying additions on the properties of alloys. We have demonstrated using the case study of Co_3_(Al,W) that one can reproduce many of the heuristically driven findings, as well as also providing a clear framework for identifying other elemental substitutions for targeted alloy properties for the next generation of cobalt based superalloys. Our work has a broader impact in that it lays the groundwork for using such informatics based methods, judiciously integrated with targeted computations, as a predictive approach for chemical design of multicomponent systems. This study has focused on exploring metrics that govern intermetallic stability and properties, but the computational framework is generic enough to integrate data from many different length scales and as such can accommodate the addition of data associated with microstructure, processing and environmental response of alloys. This will generate more complex networks and the judicious choice of appropriate algorithmic strategies will identify pathways for optimizing elemental selection to meet multiscale objectives and will be reported in a subsequent study.

## Additional Information

**How to cite this article**: Srinivasan, S. *et al.* Mapping Chemical Selection Pathways for Designing Multicomponent Alloys: an informatics framework for materials design. *Sci. Rep.* **5**, 17960; doi: 10.1038/srep17960 (2015).

## Supplementary Material

Supplementary Information

Supplementary Data

## Figures and Tables

**Table 1:** Interpretation of the graph network for Co_3_(Al,X,Y) alloys for defining new compounds with stability and at high temperatures.

Impact of alloying elements (X) in Co_3_(Al,X) as observed experimentally or suggested from first principles calculations	Comparison with informatics analysis of the impact of alloying elements (X) in Co_3_(Al,X)
Alloying Co-Al-W with Ti, V, Nb, Ta, Zr, Hf increased solvus temperature; Cr, Mn, Fe, and Ni lower solvus temperature^a^ ^24^	Ti, V, Nb, Ta, Zr and Hf are connected on the graph network pathway that enhances high temperature properties (melting point, cohesive energy) while Cr, Mn, Fe and Ni are not connected.
Alloying of Ta to Co-Al-W enhances strength at high temperature^a^ ^28^,^29^,^30^	Ta is a node on the cohesive energy directed path, suggesting an improvement of high temperature stability with the addition of Ta
Ni*, Fe*, V and Ti stabilize the γ‘ phase, while Mn and Cr do not stabilize.^b^ ^25^ Cr additions are not found to promote the stability of the ϒ’ phase^a^ ^31^*These results were for (Co,X)_3_Al, while our results are for Co_3_(Al,X).	Ti was identified as a key node on the network pathway for higher cohesive energy. V is a nearest neighbor with Ti. Mn and Cr are *not* a nearest neighbor with any node on the network pathway, suggesting that these are not expected to contribute to γ‘ stability. Our result is consistent with both experimental^31^ and computational^25^ results.
Co_3_ (Al, Nb, Mo) L1_2_ intermetallic experimentally identified and the collective addition of Nb and Mo is proposed as a substitute to W in Co_3_ (Al,X,Y) alloys^a^ ^32^	Mo and Nb are first and second nearest neighbors respectively with W in directed graph in agreement with their expected similarity in influence on high temperature stability of Co_3_(Al, X, Y)
L1_2_-Co_3_(Al_0.5_,W_0.5_) is metastable at 0K, although temperature contributions have a stabilizing effect^b^ ^33^	The graph network has clearly identified W as a strong candidate for stabilizing the L1_2_ structure. Our graph network is for design of high temperature materials and is in agreement with the initial discovery of Sato *et.al.*^1^

^a^experiment.

^b^first principles.

The interpretations of our informatics result are in very good agreement with the experimental and computational studies reported in the literature.

## References

[b1] Sato J. *et al.* Cobalt-base high-temperature alloys. Science. 312, 90–91 (2006). 16601187 10.1126/science.1121738

[b2] Suzuki A., Inui. H. & Pollock T. M. L1_2_ strengthened cobalt-base superalloys. Annual Rev. Matl. Res. 45, 345–368 (2015).

[b3] Miedema A. R., de Chatel P. F. & de Boer F. R. Cohesion in alloys – fundamentals of a semi-empirical model. Physica B+C, 100, 1–28 (1980).

[b4] Miedema A. R., Niessen A. K., de Boer F. R., Boom R. & Matten W. C. M. Cohesion in Metals: Transition Metal Alloys (North-Holland, 1989).

[b5] Villars P. Factors governing crystal structures in *Intermetallic Compounds: Principles and Practice* (eds Westbrook J. H. & Fleischer R. L.) 227–275 (Wiley, 1995).

[b6] Pearson W. Crystal Chemistry and Physics of Metals and Alloys (Wiley, 1972).

[b7] Mooser E. & Pearson W. B. On the crystal chemistry of normal valence compounds. Acta Crystallogr. 12, 1015–1022 (1959).

[b8] Seiser B., Drautz R. & Pettifor D. G. TCP phase predictions in Ni-based superalloys: Structure maps revisitied. Acta Mat 59, 749–763 (2011).

[b9] Hume-Rothery W. The Engel-Brewer theories of metals and alloys. Prog. Mater. Sci. 13, 229–265 (1968).

[b10] Balachandran P. V. & Rajan K. Structure maps for A^I^_4_A^II^_6_(BO_4_)_6_X_2_ apatite compounds via data mining. Acta Cryst. B68, 24–33 (2012). 10.1107/S0108768111054061 22267555

[b11] Broderick S. R. & Rajan K. Eigenvalue decomposition of spectral features in density of states cureves. Europhys. Lett. 95, 57005 (2011).

[b12] Broderick S. R., Aourag H. & Rajan K. Classification of oxide compounds through data mining density of states spectra. J. Am. Ceram. Soc. 94, 2974–2980 (2011).

[b13] Dey P. *et al.* Informatics-aided bandgap engineering for solar materials. Comp. Mat. Sci. 83, 185–195 (2014).

[b14] Broderick S., Ray U., Srinivasan S., Rajan K. & Balasubramanian G. An informatics based analysis of the impact of isotope substitution on phonon modes in graphene. Appl. Phys. Lett. 104, 243110 (2014).

[b15] Kong C. S., Villars P., Iwata S. & Rajan K. Mapping the ‘materials gene’ for binary intermetallic compounds- a visualization schema for crystallographic databases. Comp. Sci. Disc. 5, 015004 (2012).

[b16] Balachandran P. V., Broderick S. R. & Rajan K. Identifying the “inorganic gene” for high temperature piezoelectric perovskites through statistical learning. Proc. Royal Soc. A. 467, 2271–2290 (2011). 10.1098/rspa.2010.0543 PMC4042451 24959095

[b17] Kong C. S. *et al.* Information-theoretic approach for the discovery of design rules for crystal chemistry. J. Chem. Inf. Modl. 52, 1812–1820 (2012). 10.1021/ci200628z 22747243

[b18] Ganguly S., Kong C. S., Broderick S. R. & Rajan K. Informatics based uncertainty quantification in the design of inorganic scintillators. Matl. Manuf. Proc. 28, 726–732 (2013).

[b19] Tenenbaum J. B., de Silva V. & Langford J. C. A global geometric framework for nonlinear dimensionality reduction. Science. 290, 2319–2323 (2000). 11125149 10.1126/science.290.5500.2319

[b20] Lee J. & Verleysen M. Nonlinear Dimensionality Reduction (Springer-Verlag, 2008).

[b21] Cukierski W. J. & Foran D. J. Using betweenness centrality to identify manifold shortcuts. Paper presented at *IEEE* International Conference on Data Mining Workshops ICDMW'08, Pisa, Italy. Washington, DC: IEEE Computer Society Conference Publishing. 10.1109/ICDMW.2008.39 (2008, December 15–19). PMC2895570 20607142

[b22] Balasubramanian M. & Schwartz E. L. The Isomap algorithm and topological stability. Science. 295, 7 (2002). 11778013 10.1126/science.295.5552.7a

[b23] Wagaman A. S. & Levina E. Discovering sparse covariance structures with the Isomap. J. Comp. Graph. Stat. 18, 551–572 (2009).

[b24] Omori T. *et al.* Partition behavior of alloying elements and phase transformation temperature in Co-Al-W-base quaternary systems. Intermetallics. 32, 274–283 (2013).

[b25] Chen M. & Wang C.-Y. First-principle investigation of 3d transition metal elements in ϒ’-Co_3_(Al,W). J. Appl. Phys. 107, 093705 (2010).

[b26] Chinen H. *et al.* Phase equilibria and ternary intermetallic compound with L1_2_ structure in Co-W-Ga system. J. Phase Equilibr. Diff. 30, 587–594 (2009).

[b27] Ishida K. Recent progress in Co-base superalloy. Paper presented at 2014 TMS Annual Meeting and Exhibition, San Diego, USA. Warrendale, Pennsylvania: TMS Publications. (2014, February 16–20).

[b28] Suzuki A. & Pollock T. M. High-temperature strength and deformation of ϒ/ ϒ’ two phase Co-Al-W base alloys. Acta Mat. 56, 1288–1297 (2008).

[b29] Suzuki A., DeNolf G. C. & Pollock T. M. Flow stress anomalies in ϒ/ ϒ’ two-phase Co-Al-W base alloys. Scripta Mat. 56, 385–388 (2007).

[b30] Titus M. S., Suzuki A. & Pollock T. M. Creep and directional coarsening in single crystals of new ϒ/ ϒ’ cobalt base alloys. Scripta Mat. 66, 574–577 (2012).

[b31] Yan H.-Y., Vorontsov V. A. & Dye D. Alloying effects in polycrystalline ϒ’ strengthened Co-Al-W base alloys. Intermetallics. 48, 44–53 (2014).

[b32] Makineni S. K., Nithin B. & Chattopadhyay K. A new tungsten-free ϒ- ϒ’ Co-Al-Mo-Nb based superalloy. Scripta Mat. 98, 36–39 (2015).

[b33] Saal J. E. & Wolverton C. Thermodynamic stability of Co-Al-W L1_2_ ϒ’. Acta Mat. 61, 2330–2338 (2013).

