# Supplementary material for: Mapping Chemical Selection Pathways for Designing Multicomponent Alloys: an informatics framework for materials design
Source: Sci Rep. 2015 Dec 18;5:17960. doi: 10.1038/srep17960 (PMC4683530; doi:10.1038/srep17960)
Supplement: Supplementary Information [file srep17960-s1.pdf]

MAPPING CHEMICAL SELECTION PATHWAYS FOR DESIGNING MULTICOMPONENT ALLOYS: AN INFORMATICS FRAMEWORK FOR MATERIALS DESIGN

Srikant Srinivasan[1], Scott R. Broderick[2], Ruifeng Zhang[3], Amrita Mishra[4], Susan B. Sinnott[5], Surendra K. Saxena[6], James M. LeBeau[7] and Krishna Rajan[2**]

[1] Plant Sciences Institute-Iowa State University
[2] Dept. of Materials Design and Innovation- University at Buffalo: State University of New York
[3] Dept. of Materials Science and Engineering-Beihang University–Beijing, China
[4] Dept. of Mechanical Engineering- University of Mississippi
[5]Dept. of Materials Science and Engineering – Pennsylvania State University
[6]Dept. of Materials Science and Engineering- Florida International University
[7]Dept of Materials Science and Engineering- North Carolina State University
**Corresponding author

S1. Selection of Descriptors

We organized the descriptors into (1) parameters related to solid state properties of the constituent elements [S1-S9], (2) thermodynamic properties of the alloy chemistries using well-established models [S10-S15], and (3) electronic structure descriptors derived from the density of states (DOS) spectra from a various Co alloys. The chemistries of the DOS spectra, calculated via DFT approach [S16-S20], were selected to first include addition of elements of potential interest in Co-based superalloys, and also to include multiple alloying elements to include further chemical interactions in the database.

The initial descriptor base, when considering each value in the DOS as a unique descriptor, covers 1,045 descriptors and *a data space over 50,000 potential entries*. From this final descriptor database, covering 48 chemistries and 59 descriptors, we minimized the dimensionality of the data to increase the robustness of our approach. The developed process for selection of initial descriptor space and then the step-by-step procedure for reducing dimensionality are described in Figure S1. The selection of additive elements was chosen to include those which have been used in Ni-based superalloys. The logic is that if these elements provide beneficial properties to Ni-based alloys they may also enhance Co-based alloys. We therefore select chemical additions which may potentially enhance the alloys, while minimizing our bias in this selection.

The electronic structure description is in a spectral data format, so we employed an eigenvalue decomposition to create a discrete parameterization of the spectra without losing information. We have previously shown that representing the DOS spectra as a series of weights derived from eigenvalue decomposition captures the full complexity of the DOS [S21-S23]. The weight for each component is defined as the total DOS divided by the pattern for that component, with the patterns being constant for each compound. In this way, the weighting is proportional to the total DOS, with a constant to convert continuous data to non-continuous data.

Descriptors were calculated with both Miedema's model and DFT calculations. These calculated values were used for both selecting the direction of the pathway, as described in Figure 3, and also to validate the selection of compounds, both for enhanced properties and chemical stability. The specific roles of the Miedema values and the DFT values are described in Figure S2. This figure encompasses the entire design process, going from the initial database covering different genres of information to the reduced input data to the graph network to the pathway selection.

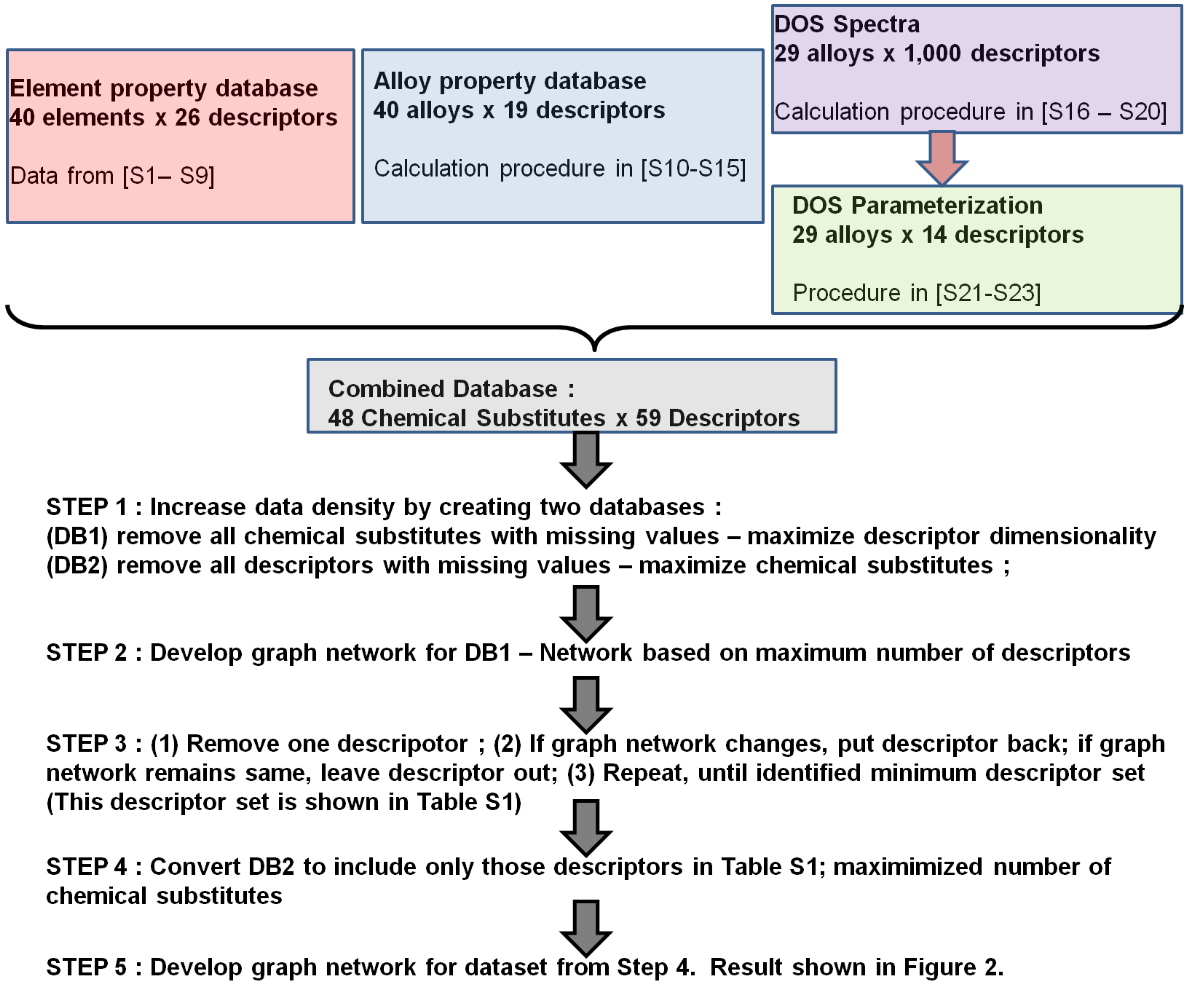

Figure S1. Process developed in this paper for selection of input descriptor base covering different data length scales, integration of the different genres of data, identification of the minimum descriptor, and development of final graph network. The data encompasses solid state elemental properties, alloy properties, and electronic structure descriptors. The initial data space includes 1,045 descriptors (26 on elements, 19 on alloys, and 1,000 on electronic structure). From this initial descriptor base, the data space (compounds times descriptors) is greater than 50,000, while in our reduced data set used to build the final graph network the data space was 880

values. This reduction increases robustness of the model, while reducing computational requirements.

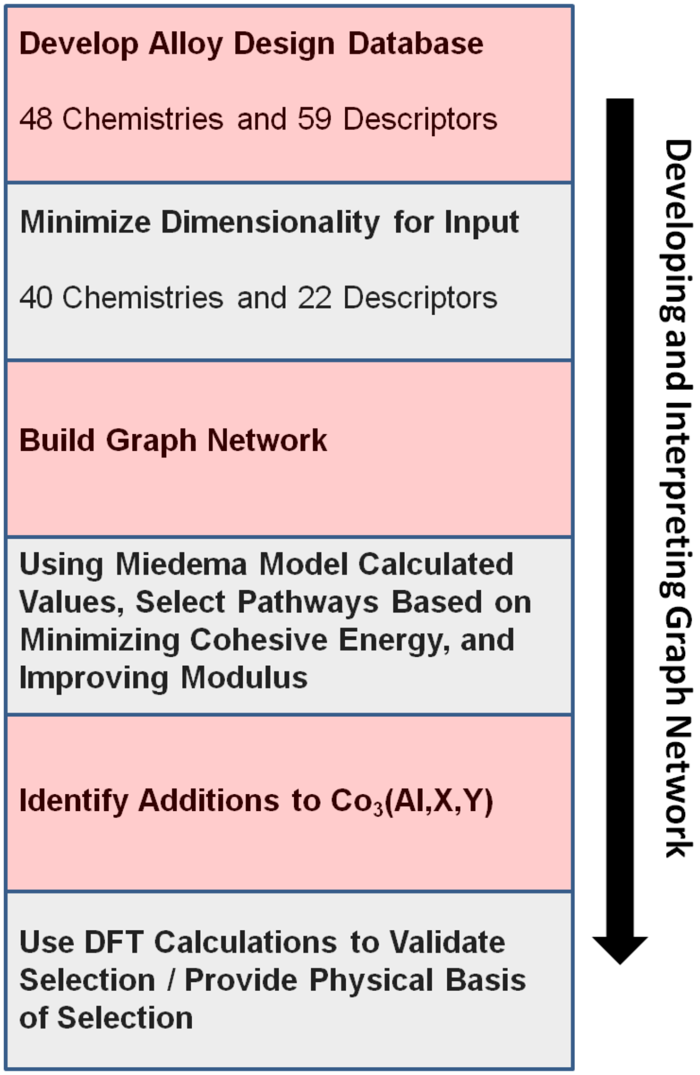

Figure S2. The role of Miedema's model and DFT for interpreting and validating the chemical selections from the graph network. From the final graph network, following the procedure of Figure S1, we select the pathway using Miedema calculation results, which we validate with selective DFT calculations. The reason for using the Miedema model for initial selection of the pathways is because the Miedema calculations are more computationally efficient and are therefore better suited for a rapid screening of chemistries.

The supplementary section is organized as follows. The final input descriptors are provided in section S2, following the procedure of Figure S1, with the description of best candidates listed in section S3. Section S4 describes the application of the Miedema model and DFT calculations, and the role of descriptors coming from each method. In section S5, we describe the development of heat maps for different data types and the interpretation of these heat maps. These heat maps provide a visual representation of the

input data and are used to identify correlations and measure of similarity in the data. We demonstrate this approach for the elemental descriptors, and therefore represent the periodic table in this alternate representation. In section S6, we describe the development of alloy property descriptors, combining various well-established models in the materials science field. The development of the electronic structure parameterization from the density of states spectra is described in section S7. The mathematical details of the Isomap approach used for developing the graph network is discussed in section S8, while section S9 highlights some key features of the graph networks. Finally in section S10 we discuss the results of our DFT calculations, which we use to verify the selection of pathways in the graph network, which are initially defined via Miedema's model, as described in Figure S2.

S2. Selection of Final Input Descriptor Set

The final descriptor set was determined using the procedure described in Figure S1. While we have developed a large database with properties applicable to the problem, an additional step we performed prior to manifold analysis to ensure robustness of analysis. We first remove redundancy from the data set. The reason for doing the descriptor reduction is to remove any bias by over-impacting the analysis by including the same information an overly large amount of time, and also by reducing the descriptor set the future data creation requirements are reduced. Second, we need to ensure high data density and therefore perform the analysis where we are able to have results with high accuracy. By considering these data requirements, we ensure high robustness and accuracy of the models, while making the models able to more easily encompass future data and materials, and more effectively defining data gaps.

The input descriptor set utilizes the database described while making the final descriptor selections based on the considerations discussed above. The final requirement is to ensure that all of the property categories that we defined as design requirements are represented in the analysis. All of these data requirements are addressed in our input descriptor set, as shown in Table S1. These descriptors incorporate the various descriptor length scales, the design categories, and include descriptors within high data density and encompassing a large number of chemical substitutes. These descriptors contribute to a robust, accurate and interpretable analysis for alloy design.

Table S1. Input descriptor space for manifold analysis. These descriptors address the primary design requirements in electronic property, high temperature strength, structure, size and lattice coherency, and thermal expansion. This set of descriptors incorporates the different components of our database as discussed in the previous sections, with the data collected and prepared from the previously mention references [S1-S23]. The final selection of descriptors was based on minimizing data redundancy thereby improving robustness, increasing data density and improving accuracy, and increasing the number of chemistries that can be included in the analysis.

Electronic Property	Martynov-Batsanov electronegativity
	Work function
	Pauling electronegativity
	First Ionization Potential
High Temp Strength	Shear moduli
	Bulk moduli
	Electron-density mismatch
	Melting point
	Cohesive energy
	Enthalpy of Formation
Structural Factors	Density
	Atomic number
	Atomic weight
	Pseudopotential core radii sum
Size Factors	Covalent Radius
	Volume Change
	Molar volume
	Lattice mismatch
Thermal Factors	Coefficient of Thermal Expansion
	Specific Heat
	Thermal Conductivity
	Electrical Conductivity

S3. Graph Theory Mathematical Details

S3.1: Graph construction in R^n

In order to construct the initial graph in R^n the popular options are: K nearest-neighbors (KNN) and the ϙ-ball. In KNN graphs the each point is connected by an edge to its $'k'$ nearest neighbors alone $\ni\ d_{ij} = \infty,\ \forall\ |i - j| > \mathrm{k}$. In the ϙ-ball method, on the other hand, one picks a fixed Euclidean radius around every point x_i and connects all points within this radius such that $d_{ij} <$ ϙ. In both cases, the critical parameters (k and ϙ) can be thought of as tuning parameters and depend on the problem, thus forming a semi-supervised process. The former can lead to disconnected graphs in the absence of edges connecting every vertex to every other one while latter case is sensitive to local scale and can poor graph connectivity in data with varying density. Among semi-supervised learning KNN has been found to perform well compared to other graphs [S24] and was, therefore, employed in the present work. In this work the choice of k was optimized by statistically determining the smallest value that could minimize the residual variance $|\ d_M\ -\ d_G\ |$, while providing the maximum number of alternative paths. This ensures that the resulting graph is neither over-connected, leading to loss of pairwise geodesic distances, nor are critical neighbors disconnected. For each data point, we also compute the ratio of the distance to its closest and farthest neighbor. The ratios are then averaged over all data points to calculate a scale-invariant, global parameter, Δ, [S25] to estimate the measure of uncertainity introduced by sparsity in high dimensional spaces, given that the data points must have sufficient density on the manifold [S26]. Δ can range between zero and one and a small value indicates a healthy variance in pairwise distances.

S3.2: Computing geodesic distances from M^d

Once the graph is constructed the geodesic distances d_G between elements x_i and x_j for $|i\text{-}j|>k$ is estimated by calculating the pairwise euclidean distances along the edges connecting all the intermediate vertices between x_i and $x_{j,}$ and applying Floyd's algorithm. To do so we initialize $d_G = d_{ij}\ ;\ |i\text{-}j|<k$ and $d_G = \infty$ otherwise. Then for each value of $k = 1, 2, \ldots, N$ in turn, replace all entries $d_G(i,j)$ by $min\{d_G(i,j), d_G(i,k) + d_G(k,j)\}$. The matrix of final values $D_G = \{d_G(i,j)\}$ will contain the shortest path distances between all pairs of points. Finally the dimensions of M^d are recovered by centering the square of the distance matrix D_G and computing its eigen values (λ_g) and eigen vectors (V_g). The new axes of the low dimensional embedding as defined as $\sqrt{\lambda}V$. These new axes represent the dimensionally reduced submanifold and the elements y_i plotted out on these new axes form the vertices of the weighted graph whose edges representthe geodesic distances.

Summary: The Isomap stepwise procedure involved in the mapping $x_i \longrightarrow y_i$ sequentially comprises:

i) Compute the matrix of Euclidean distances [E] between each pair of points (x_i, x_j) in R^n.
ii) Retain only those entries of [E] which connect every point to its 'k' nearest neighbors to obtain the reduced matrix [E_k].
iii) Compute the matrix of Geodesic distances [G] by approximating with the graph distances between each pair of points (x_i, x_j) in R^n from [E_k] using Floyd's algorithm.

iv) Compute the dissimilarity matrix [A] which is obtained by centering the Hadamard square of [G] with the Gram matrix [H].

v) Solve the Eigen value problem for [A] to obtain the eigen pairs (λ, **v**). The product of the eigen pairs $\sqrt{\lambda}V$ provide the low dimensional embedding of the non-linear manifold.

S4. Minimizing Short-Circuit Errors in Graph Network Manifold

The optimal number of dimensions in which to represent the graph output of Isomap can be determined by a Scree plot (Fig. S3) which is an ordered representation of the impact of each additional dimension, in the low dimensional representation, in accurately representing the geodesic distance along the original manifold. Since the manifold in high dimensional space can vary depending on the number of nearest neighbors chosen, a measure of statistical uncertainty in the geodesic distances can be obtained by varying the number of nearest neighbors to check for short-circuit errors as well as by ensuring the optimum number of dimensions for low dimensional representation. In this case we are defining uncertainty in terms of the sensitivity in the identified connections to the input parameters of neighborhood size and number of dimensions. For this reason we have explored the relationships and pathways for various nearest neighbor values and with changing dimensions. Specifically, we modify the input parameters of number of nearest neighbors and the number of dimensions and information captured by each respective Isomap. By defining uncertainty in this way, we ensure we do not short circuit the pathway. This follows from the comments of Balasubramanian and Schwartz [S26] that the connectivity of each data point is sensitive to short-circuiting depending on the neighborhood size and the manifold, thereby impacting the entire geodesic distance matrix. The considerations thus for ensuring that we do not have this short-circuit error is thus the neighborhood connections, and dimensions which impact the manifold. For this reason, we have explored the sensitivity of the pathways to ensure that our results are not a function of the parameters. After careful inspection, we find that our conclusions are consistent even with changing parameters and we are not introducing short-circuit errors.

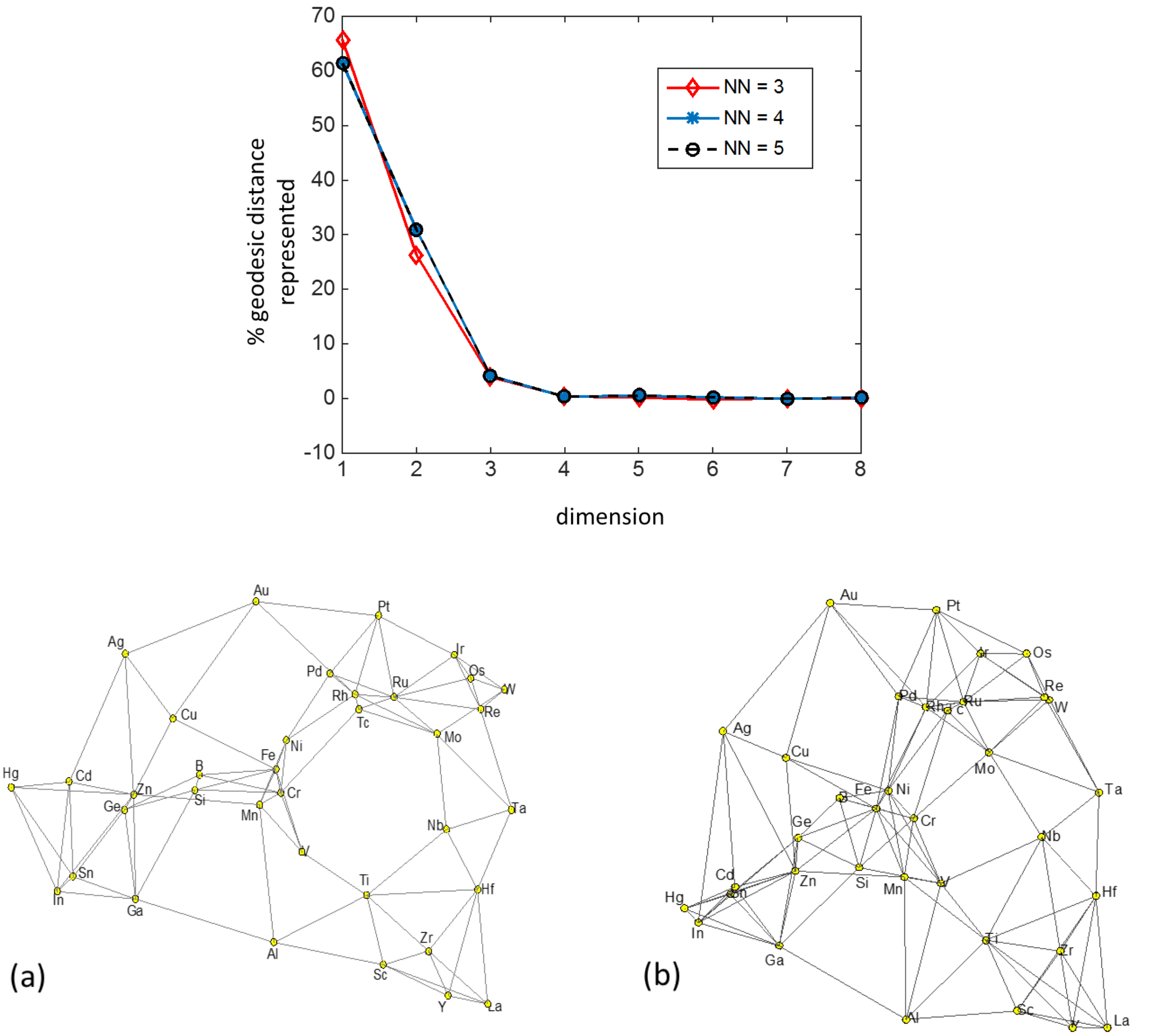

Fig. S3: Scree plot showing that the first two dimensions are accurate in representing 90% of the geodesic distance in the high dimensional manifold. The increase in number of neighbors does not significantly alter the results. Comparison of the graph networks (a and b) for four versus five nearest neighbors provides a measure of uncertainty. In no cases do we see a change in the connections, demonstrating that the uncertainty in our results here is low.

S5. Ranking of Best Substitutes for $Co_3(Al,X,Y)$

In Fig. 4, we provided a graph network with connections between elemental substitutes defining similarity in compounds, in terms of their application to stabilizing Co_3Al. From this network, we identify degree of similarity to W, thereby identifying additional substitutes for Co_3(Al,X,Y). Table S2 provides a listing of the best candidates, based on number of connections needed to get to W. The best candidates are those that have a direct connection to W (Mo, Re, Os). We therefore identify the best alloy candidates are Co_3(Al,X,Y), with X,Y = W, Mo, Re, Os. The next best candidates are those that are connected to Mo, Re, and/or Os, and therefore are two connections from W. For example, Ta is connected to Re, which is connected to W. These two-level connections provide additional candidates for chemical substitutes. In fact, Co_3(Al,W,Ta) has been shown to exist in a stable phase with the Ta addition further improving properties [S25]. Therefore, these other second tier candidates have high potential as chemical additives, although we propose the W nearest neighbor elements as being even better. The third nearest neighbors (for example, Hf is connected to Ta which is connected to Re which is connected to W) may be suitable for additives. They likely due not contribute in stabilizing. However, these elements may contribute other beneficial properties with minimal affect on stability. Therefore, this table reduces and ranks the potential substitute space for designing higher order Co-based alloys.

Table S2. Ranking of alloy substitutes for Co_3(Al,X,Y), based on number of connections to W in Fig. 4. For example, Mo, Os, and Re are directly connected to W, and therefore these three and W are predicted to have the best properties as chemical substitutes. The next tier of candidates requires connection through a node to get to W, and therefore are also potential substitutes, but are predicted to be less effective than the W first nearest neighbors. The addition of Ta has been shown to improve Co_3(Al,W) [S27], thereby providing a basis for our substitute identifications.

**Best Candidate For Addition**	**Second Tier Candidates**	**Third Tier Candidates**
(1 connection to W)	*(2 connections to W)*	*(3 connections to W)*
Mo	Ir	Hf
Os	Nb	Pd
Re	Rh	Pt
	Ru	Ti
	Ta	
	Tc	

S6. Application of Miedema's model and DFT calculations

To capture similarity beyond the general classification based on elemental descriptors, the formation enthalpies and cohesive energies of Co_3(Al,X) compounds are introduced. We adopt a two pronged approach for inclusion of thermodynamic data: one is the application of Miedema's model for rapid generation of relevant parameters in the exploratory phase. Further, the Miedema parameters are used for guiding the directed pathways, with selective use of DFT calculations for validation of the selected pathways.

The formation enthalpies contributing to the input are calculated by Miedema's model which can be expressed with element B as the solute by [S2-S5,S7-9]:

$$\Delta H_{\text{Mied}}^{\text{for}} = f(\text{c}) \cdot \frac{S(\text{c}) \cdot V_{A}^{2/3}}{\left(n_{ws}^{-1/3}\right)_{av}} \cdot \left\{-P(\Delta\Phi^{*})^{2} + Q\left(\Delta n_{ws}^{1/3}\right)^{2}\right\}$$

where f(c) is a function of alloy composition, which has considered the effect of the chemical short-range-order (CSRO) in an ordered intermetallic compound on the formation enthalpy, and is expressed by $f(c) = c_A^s \cdot c_B^s\{1 + \gamma \cdot (c_A^s \cdot c_B^s)^2\}$, where γ is the CSRO parameter for the intermetallic compound and is taken as a constant, i.e., γ= 8. The pre-factor S(c) is defined to describe the effect of the atom size difference on the contact interface and the bonding energy and is expressed as $S(c) = 1 - c_B^s \left|V_A^{2/3} - V_B^{2/3}\right| / \left\{c_A^s V_A^{2/3} + c_B^s V_B^{2/3}\right\}$. Note that when A is the solute component $S(c) = 1 - c_A^s \left|V_A^{2/3} - V_B^{2/3}\right| / \left\{c_A^s V_A^{2/3} + c_B^s V_B^{2/3}\right\}$. As $\left|V_A^{2/3} - V_B^{2/3}\right|$ is an absolute value and therefore is always positive, the pre-factor S(c) is a value within a range of 0 < S(c) < 1. When S(c)=1, equation (1) becomes the original Miedema's model. The cohesive energy using Miedema's model is then calculated as:

$$E_c^{\text{Mied}}(A_x B_{1-x}) = x. E_c(A) + (1 - x)E_c(B) - \Delta H_{\text{Mied}}^{\text{for}}(A_x B_{1-x}).$$

Since these equations relate to binary systems, the binary properties are calculated and from these the ternary properties are determined again as a scaling of composition. For $Co_3(Al,X)$, the formation enthalpy and cohesive energy were calculated as a function of those for CoAl, CoX and AlX.

In order to validate our selected design pathways, additional DFT calculations were performed. Our ab initio total-energy and force calculations based on DFT were implemented in the Vienna ab initio simulation package (VASP) [S10-S11]. A gradient corrected functional was used to describe electronic exchange and correlations [S12]. Electron-ion interactions were treated within the projected-augmented-wave (PAW) method [S13-S14] with Perdew-Burke-Ernzerhof (PBE) for the exchange-correlation functional. A convergence criterion in the total energy of 500 eV kinetic energy cutoff and with a (15×15×15) k-point mesh was used.

S7. Data mapping

We use heat maps here to visualize our data so that correlations, data gaps, and outliers are easily visualized and the pair-wise correlations between chemical substitutes are mathematically defined. The heat map of the elemental descriptors is shown in Figure S4 and captures the mulit-colinear relationships between descriptors, as well as a correlated dendrogram, which describes degree of correlations. This particular heat map provides a representation of the periodic table, where the magnitude of each property value is represented by changing color, and the property are for the single elements. By considering single element properties, we are able to map the influence of elements on alloy properties.

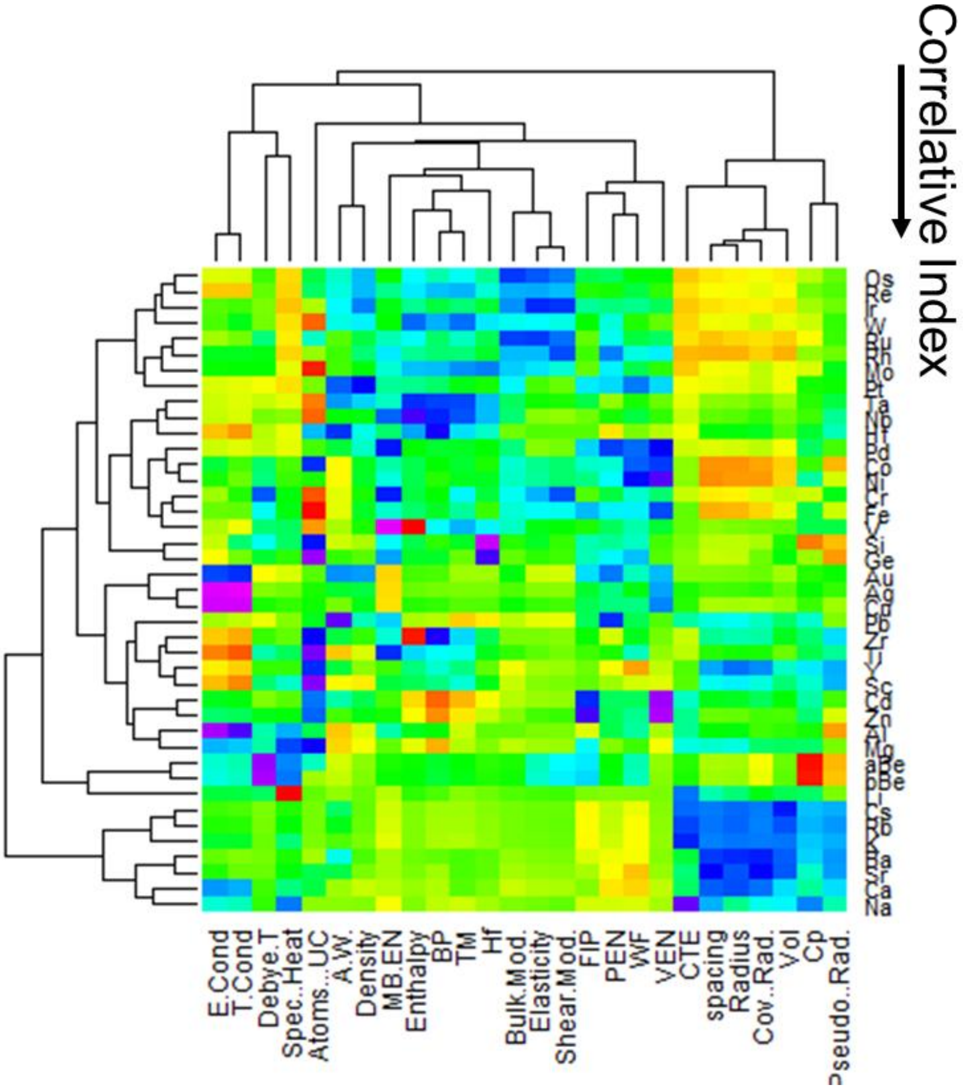

Figure S4. Heat map and associated dendrograms capturing the pair-wise correlations between descriptors associated with single elements. The purpose of employing the heat map is to ensure in the final descriptor selection we are including descriptors which are not strongly correlated (ie. sampling from the different "branches") and are therefore assessing a diversity of descriptor correlations and physics.

The dendrograms define the degree of correlation, whether strong, fuzzy or weak. The closer the connecting branch is to the map (ie. higher correlative index) then the higher the degree of correlation. The identification of descriptor correlations provides some guidance for targeted chemical substitutions. This demonstrates that the heat map captures the complexity of the correlations and serves as a suitable input into the Isomap algorithm

The objective of the Isomap algorithm is to map the distribution of elements in the high dimensional space, represented by the set of data points $\{x_i\} \in R^n$, onto a convex nonlinear manifold M^d of lower

dimension $d < n$ and through dimensionality reduction, obtain a two or three dimensional embedding of the elements into a weighted graph. The mapping is carried out such that the geodesic distances between the elements in the higher dimensional manifold is preserved when it is mapped onto the lower dimensional graph, so that the edges of the graph are weighted in their length according to the original geodesic distances. The dissimilarity between alloying elements, which themselves form the vertices of this graph, are captured by these distances between them along the edges that connect them to their nearest neighbors. This mapping can be described in set theory as: $x_i \rightarrow y_i \mid y_i \in M^d$, $d<n$, *s.t.* $\forall(i,j): \left|x_i - x_j\right|_\beta = \left|y_i - y_j\right|_\beta$ where β is a norm, representative of the pairwise geodesic distances d_{ij} between any two elements $'i'$ and $'j'$, which is the curvilinear distance along the manifold in M^d.

S8. Development of Alloy Property Descriptor Base

The deriving of alloy properties is based on the well-established rules such as from Villars (eqs. S1 and S2), Mooser-Pearson (eqs. S3 and S4), and Pettifor (Equations S5 and S6). The use of the Miedema model provides calculations of pair-wise interactions between elements, with the equations shown in eqs. S7 and S8. These properties are based on the linkage between the descriptors of the elemental constituents and the alloy properties.

$$\overline{\Delta X^{MB}} = \sum c_i X_i^{MB} \quad \text{(S1)}$$

$$\overline{\Delta R_{s+p}^2} = \sum c_i \Delta R_{s+p\ i}^2 \quad \text{(S2)}$$

The use of Villars rule for defining the electronegativity of the alloy is ref. S1. The concentration is represented in eqs. S1 through S5 by the term c_i. In this way, we can integrate composition into the analysis as well, where the ratio of Al to X in $Co_3(Al,X)$ is defined by this term. In this work, we assume site substitution of the Al site. While the use of these equations can allow for the consideration of compositional variations, we instead focus on a consistent composition with addition of elements. Further, while these equations are expandable to any number of elements, our calculations have been performed for one or two substitutions only.

$$n_{av} = \sum c_i n_i \quad \text{(S3)}$$

$$\overline{\Delta X^{Pauling}} = \sum c_i \Delta X_i^{Pauling} \quad \text{(S4)}$$

$$\overline{N} = \sum c_i N_i \quad \text{(S5)}$$

$$\frac{\overline{\Delta V}}{V} = \sum c_i c_j \frac{2|V_i - V_j|}{V_i + V_j} \quad \text{(S6)}$$

In the case of volume expansion, the change with chemical substitution is based on the differences in the elemental values. If we assume element X substituting for Al in $Co_3Al_{0.8}X_{0.2}$, then the volume expansion

is based on the size of element X and Al, with $c_i = 0.2$ and $c_j = 0.8$. This calculation is then repeated for every possible element X, or combination of elements so that the volume expansion or lattice misfit is calculated for every substitution, up to ternary substitutions. As with the other cases, this demonstrates the connection between elemental properties and alloy properties. However, the variety of descriptors serving as chemical substitutes demonstrates the need for informatics analysis to reduce the multi-criteria design at the different length scales so that basic chemical design rules are teased out.

$$\Delta\varphi^* = \Delta\varphi^*_{AB} + \Delta\varphi^*_{BC} + \Delta\varphi^*_{AC} \quad \text{(S7)}$$

$$\Delta n^*_{WS} = \Delta n^*_{WS_{AB}} + \Delta n^*_{WS_{BC}} + \Delta n^*_{WS_{AC}} \quad \text{(S8)}$$

The application of Miedema's rules provide integration of pair-wise interactions which are calculated very rapidly with the more complex alloy chemistries. In the case of binary compositions, the work function and Wigner-Seitz cell density are rapidly calculated. If we expand these binary relations to more complex chemistries, the values are then calculated as these values scaled by the composition. This accounts then for the interactions of chemistries. This then advances beyond the characteristics and limitations of the periodic table by accounting for the weightings of elemental properties in different amounts, as well as the change in interactions. These interactions are also further expanded upon in our addition of crystallographic descriptors. The representation we use for the alloy properties, partially represented in the above equations provides a basis for linking elemental additions with the overall material performance with minimal computational requirements. The data developed associated with the alloy characteristics is shown in Figure S5.

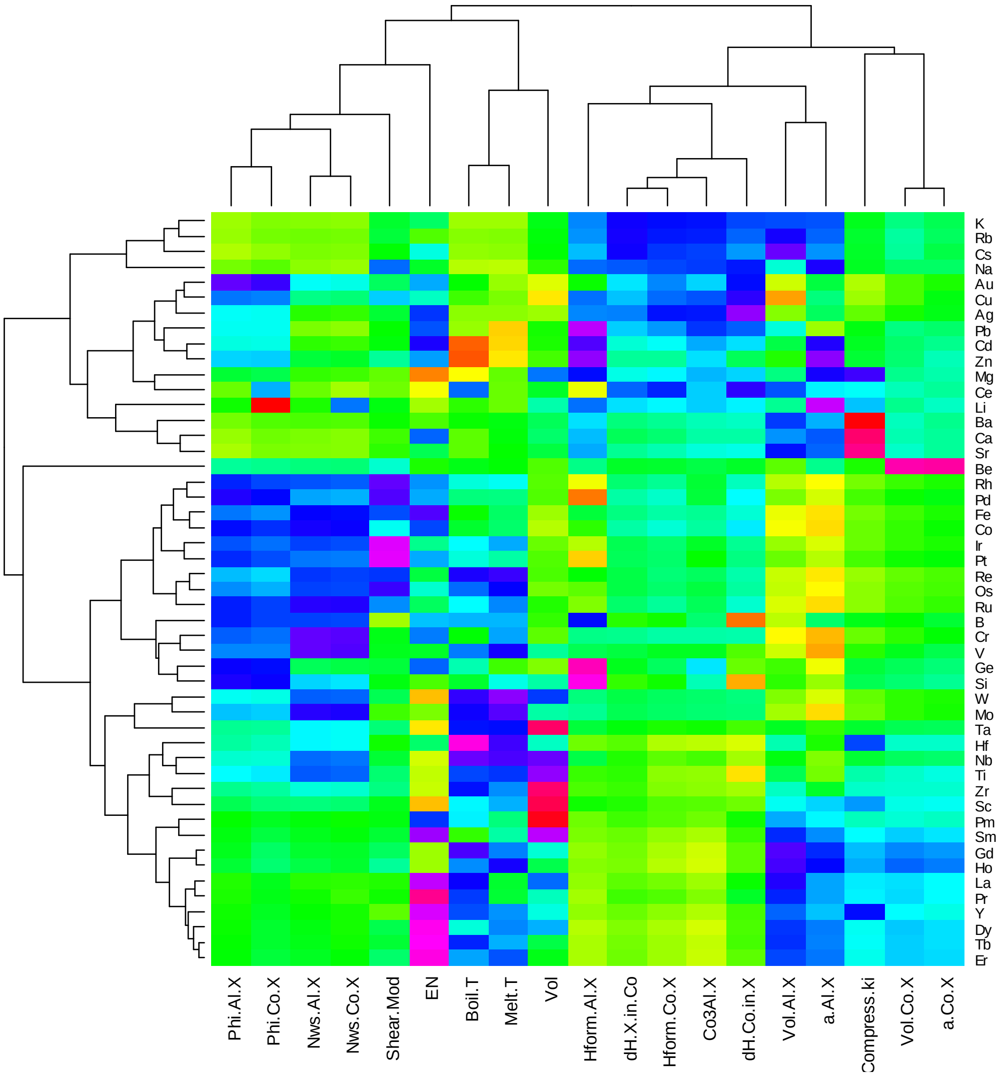

Figure S5. Heat map for alloy properties based on developing the descriptors from the models of equations S1 through S8, and through use of Miedema's model. The descriptors include lattice mismatches, both through the change of volume and also the change in volume as a function of site occupancy, as calculated through Miedema's model. The change in enthalpies, as well as correlative electronegativities and electron orbital interactions are represented within this plot. High temperature character is represented in this data through calculated melting point. The inclusion of these characteristics ensures that the extension of elemental characteristics to alloy behavior is represented in our analysis.

S9. Development of Electronic Structure Descriptor Base

The electronic structure descriptor space was based on an analysis of the various DOS spectra, with the objective of developing a parameterization of the spectra with minimal introduction of redundancy or noise. These DOS spectra were calculated via density functional theory calculations. The process for the parameterization of these spectra is shown in Figure S6. The series of DOS curves were analyzed with principal component analysis, and the curves were decomposed into the primary patterns differentiating the curves and the parameterization of these patterns required to reconstruct the original spectra. The values input into the data analysis were these weights for each alloy with fourteen different weights. For example, the DOS for Co_3Al can be reconstructed as the sum of the weights multiplied by the corresponding spectral patterns (Figure S7). The final spectra are the sum of the fourteen spectra after the reconstruction, as well as mean corrections. These parameters associated with each chemical substitution were then used to develop the heat map related to electronic structure. In this process, the patterns were defined as being orthogonal to each other, and in that way no redundancy in data was introduced. The parameters link the contribution of these various patterns with the overall DOS. By including these parameters in the analysis, the contribution of the elemental descriptors on the electronic structure, as well as the impact of changing electronic structure on the alloy properties, is integrated into the analysis. The parameterization of the DOS spectra is described in our prior works [S21-S23]. This work then first integrates different data types (spectral with non-continuous values), while providing an input for an analysis to discover the best chemical substitutions while incorporating multiple length scales and the complex interactions between the length scales. The heat map showing these electronic structure parameters for each compound are shown in Figure S8. Of note, since the weightings are each associated with different principal components, and since the principal compounds are orthogonal to each other, the weights each provide unique information. This is represented by no connections in the dendrograms associated with the weights. Also, since DFT calculations were performed for both single and binary substitutions, alloys of different complexities are represented in the heat map.

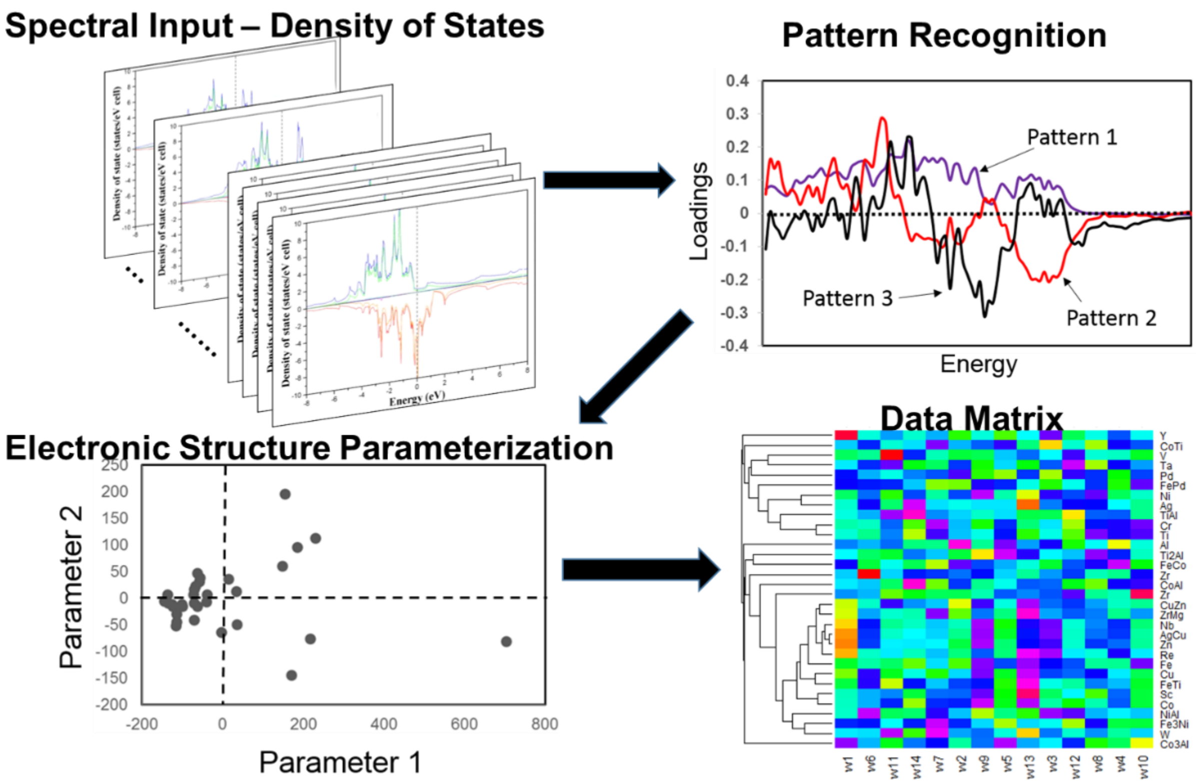

Figure S6. Converting the spectral data representation of the DOS spectra into a parameterization which captures the relationships with minimal effects due to redundancy or noise in the data. By first maximizing the signal corresponding with unique information and then providing a minimal number of values required to describe the DOS, we are able to integrate this type of analysis without over weighting of its impact into our chemical design methodology.

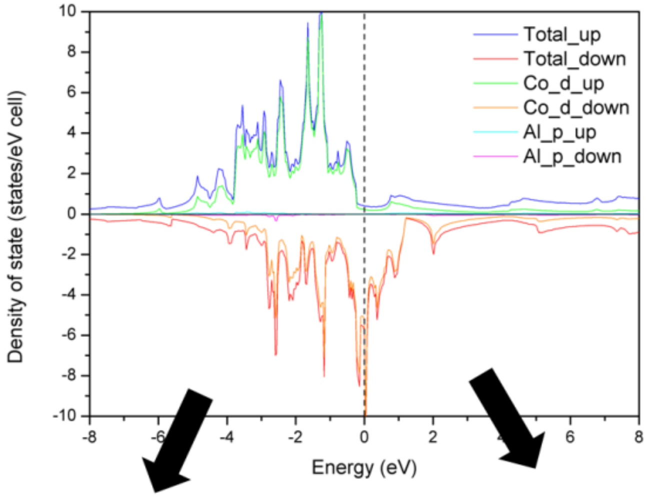

$$DOS = \sum_{i=1}^{14} WiPi$$

Co_3Al	
W1	154.9
W2	195.4
W3	76.3

•

•

•

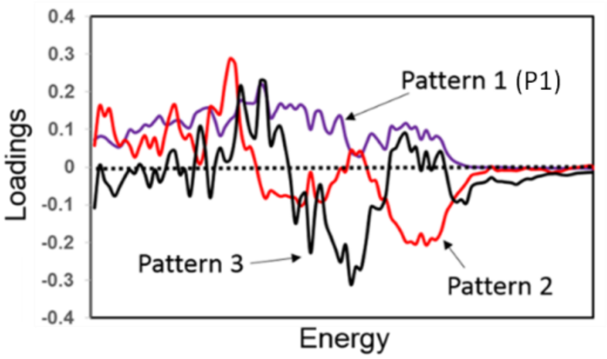

Figure S7. Relationship between weights (W) and total DOS. In Figure S5, we showed the process for identifying the weights. This figure defines the correlation, with the first three weights of a total of fourteen weights for Co_3Al shown. Unique weights were calculated for each chemistry. A total of fourteen patters (P) were also calculated, with the patterns applicable for every chemistry. The total DOS is then defined as the sum of the weights times the patterns.

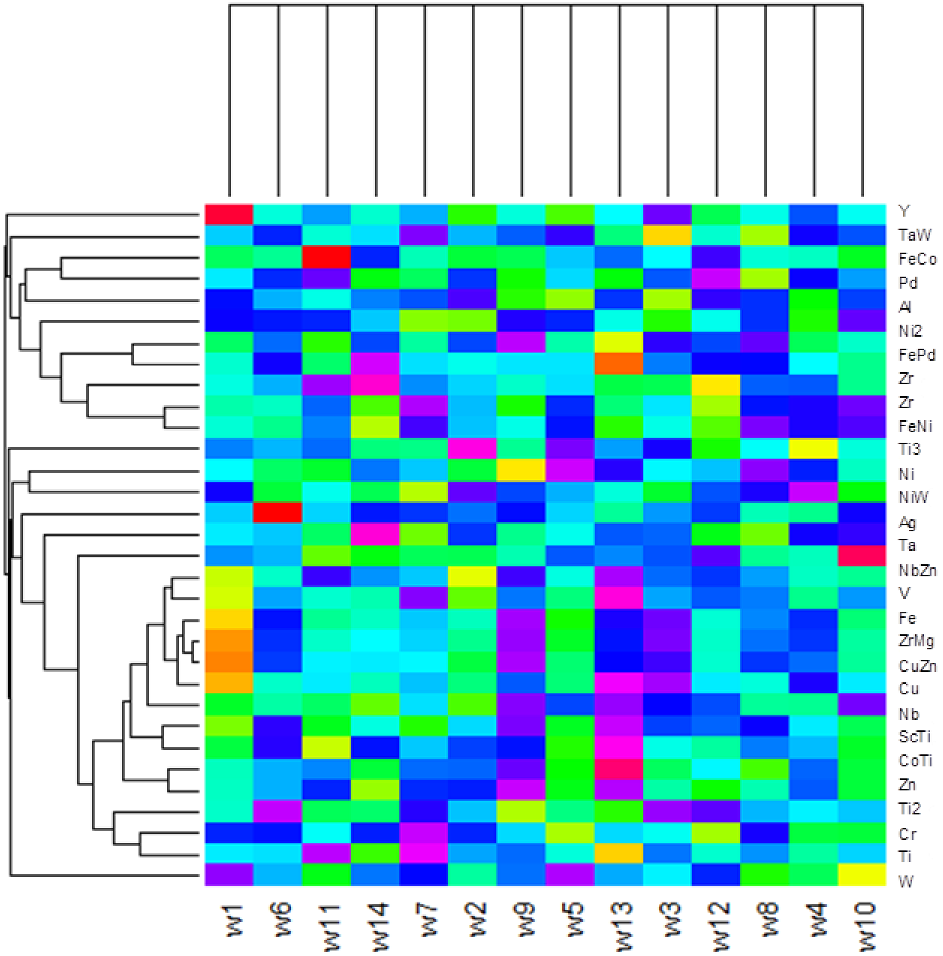

Figure S8. Heat map representing the electronic structure of the alloys, where the weights (w) correspond to the importance of specific spectral patterns to the overall DOS spectra. The input DOS spectra were calculated via DFT calculations, and provide comparison in subtle features in the DOS that otherwise would not be identified. The DOS spectra for all these alloys have been reduced from over 1,000 points per spectra to 14 points with zero loss of information. The reduction in parameters allows the electronic structure descriptors to be integrated into the analysis without biasing the analysis by having too many associated descriptors.

S10. Dissimilarity graph representation of $Co_3(Al,X)$

We provide a magnified view of the dissimilarity graph for the set of elements (X) in $Co_3(Al,X)$ in Fig. S9. As noted in the text, this graph has a 3-D component which is projected onto a 2-D plane. Therefore, it might obscure connections between vertices or appear that there are intersections between the edges where there aren't any. For example, in the lower left section there is a separate edge connecting {In, Sn}, {Sn,Ga} and {In, Ga} although it appears that they are in a straight line. Similarly, in the top right image there is an edge connecting the pair of vertices {Mo,W} that appears to intersect with {Re, Ta}, but these are non-intersecting in a 3D version. Further, there is a direct pathway between Mo and Re, which does not clearly show due to the 3D nature of the network. Sections where such clustering of vertices may occur are blown up for clarity.

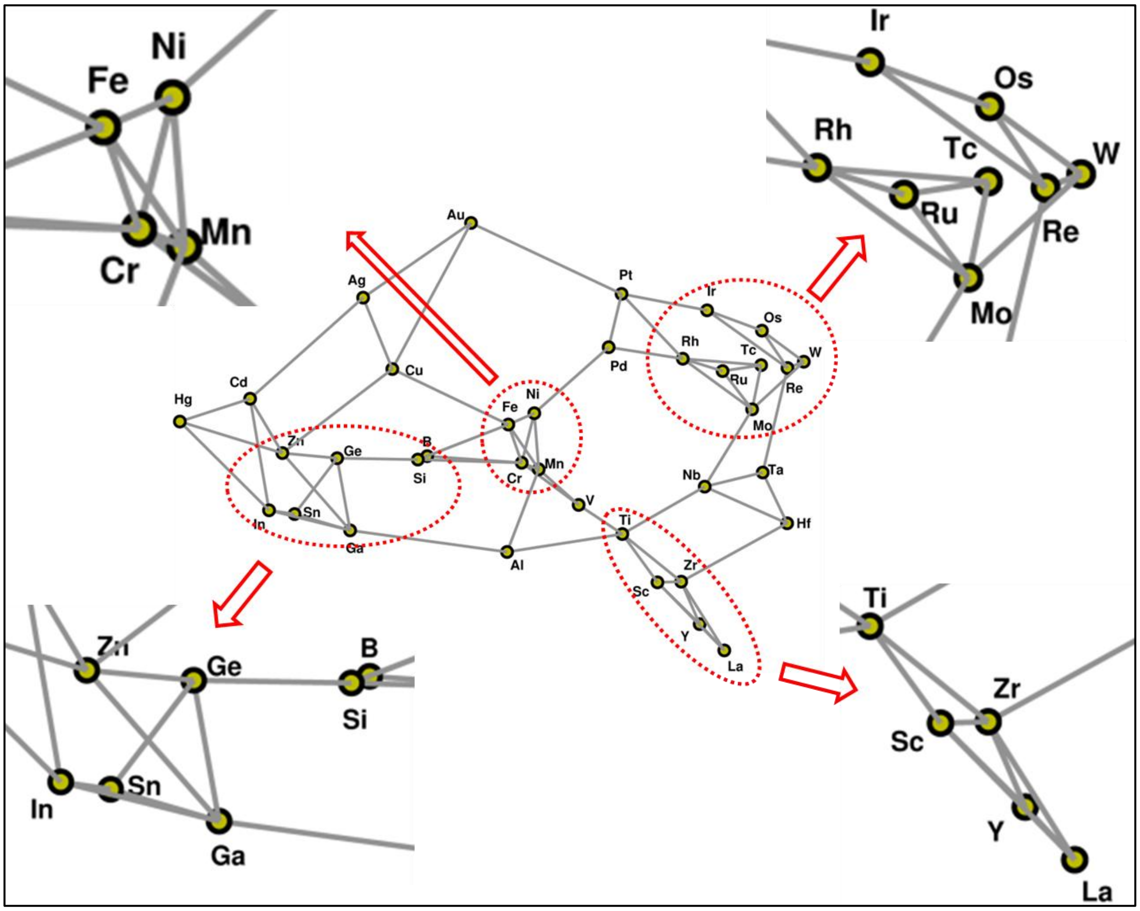

Figure S9: A magnified view of subnetworks within the dissimilarity graph to enhance interpretation of interconnection between the different vertices. Due to the high density of points within some regions of the network as well as the 3D nature of the graph, the connections of some nodes are less clearly visualized in this representation, although mathematically our analysis captures these fuzzier connections. It should be noted that there is a 3-D component to the graph in Fig. 2 and the distances can potentially be misinterpreted. For example, B may

appear closer to Fe than Mn. However, in a rotated 3-D view B sits above the plane containing Fe and Mn.

S11. DFT Calculations for Identifying Design Pathways

To quantify the similarity of Co_3Al (unstable) with effects due to additives which enhance stability, the density of states (DOS) was calculated for chemistries following both the bulk modulus and cohesive energy pathways, while the enthalpy of formation, and cohesive energy were calculated for all chemical substitutes in the $L1_2$ structure. The role of DFT here is to validate the pathways taken within the graph network for identifying the stable chemistries and to assess the physics of our network. For graph traversal we had calculated the cohesive energy and enthalpy via Miedema's model. Both the resulting graph network and selection of design pathways has qualitatively the same result using either DFT or Miedema's model. Of the additives that we identified for modifying Co_3Al, the results are as follows: Co_3Ta has a comparable cohesive energy to Co_3W, while Co_3Ti possess a high thermal stability due to the most negative formation enthalpy (Fig. S10). Fig. S11 shows the calculated density of state (DOS) of $L1_2$-Co_3X, with X = Al, W, Ta, Ti and Mo. Clearly seen is that Co_3Al shows similar topological electronic structure with spin up DOS, but difference in spin-down DOS, while Co_3W and Co_3Mo are less similar to Co_3Al. This result is in agreement with our graph network which found that the addition of Ti had the least effect on $Co_3(Al,X)$, while the addition of Mo or W resulted in more significant change. For Co_3Ti the peak near the Fermi energy (E_F) is smeared, suggesting a high stability. The similarity of $L1_2$-Co_3W and $L1_2$-Co_3Mo can also be seen from the calculated density of states. The Fermi level is located near the shoulder at the pseudogap, while it is located at the pseudogap for Co_3Mo, in agreement with the more negative formation enthalpy for Co_3Ta as compared with Co_3W. This result suggests that the introduction of Mo may further stabilize the $L1_2$-$Co_3(Al,W)$ compound.

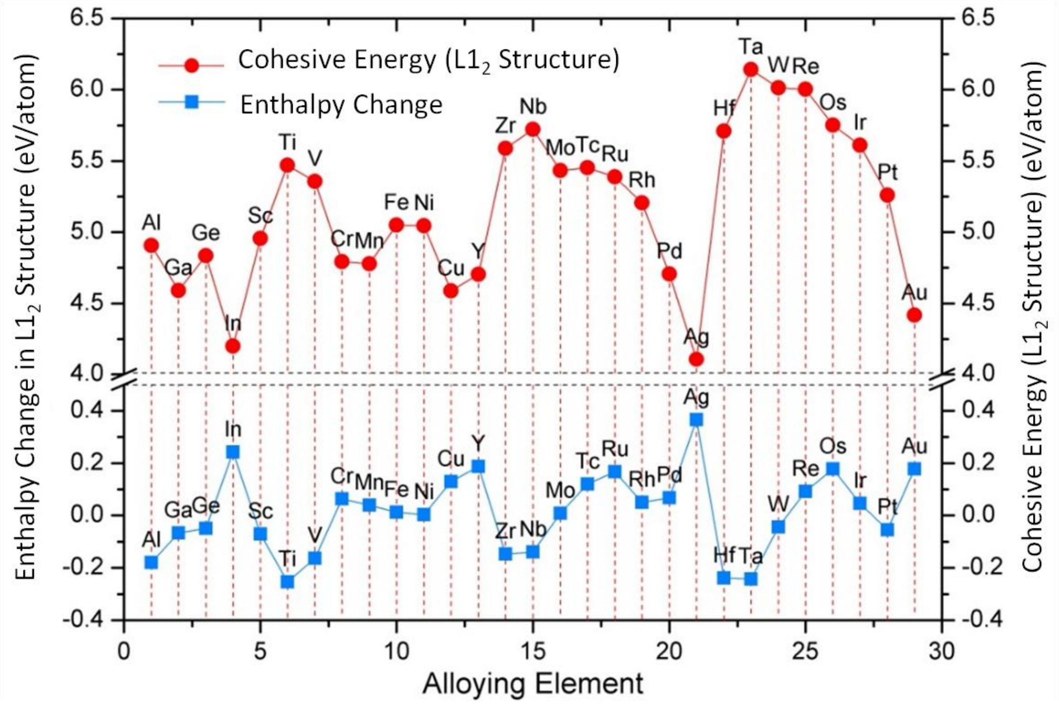

Fig. S10. DFT calculation of formation enthalpies and cohesive energies of L1$_2$-Co_3X. These results indicate Co_3Ta and Co_3Re have a comparable cohesive energy to Co_3W, while Co_3Ti, Co_3Hf and Co_3Ta possess a high thermal stability due to the most negative formation enthalpy (without consideration of lattice stability of different structures, e.g. L1$_2$, D0$_{19}$, D0$_{22}$, D0$_{24}$).

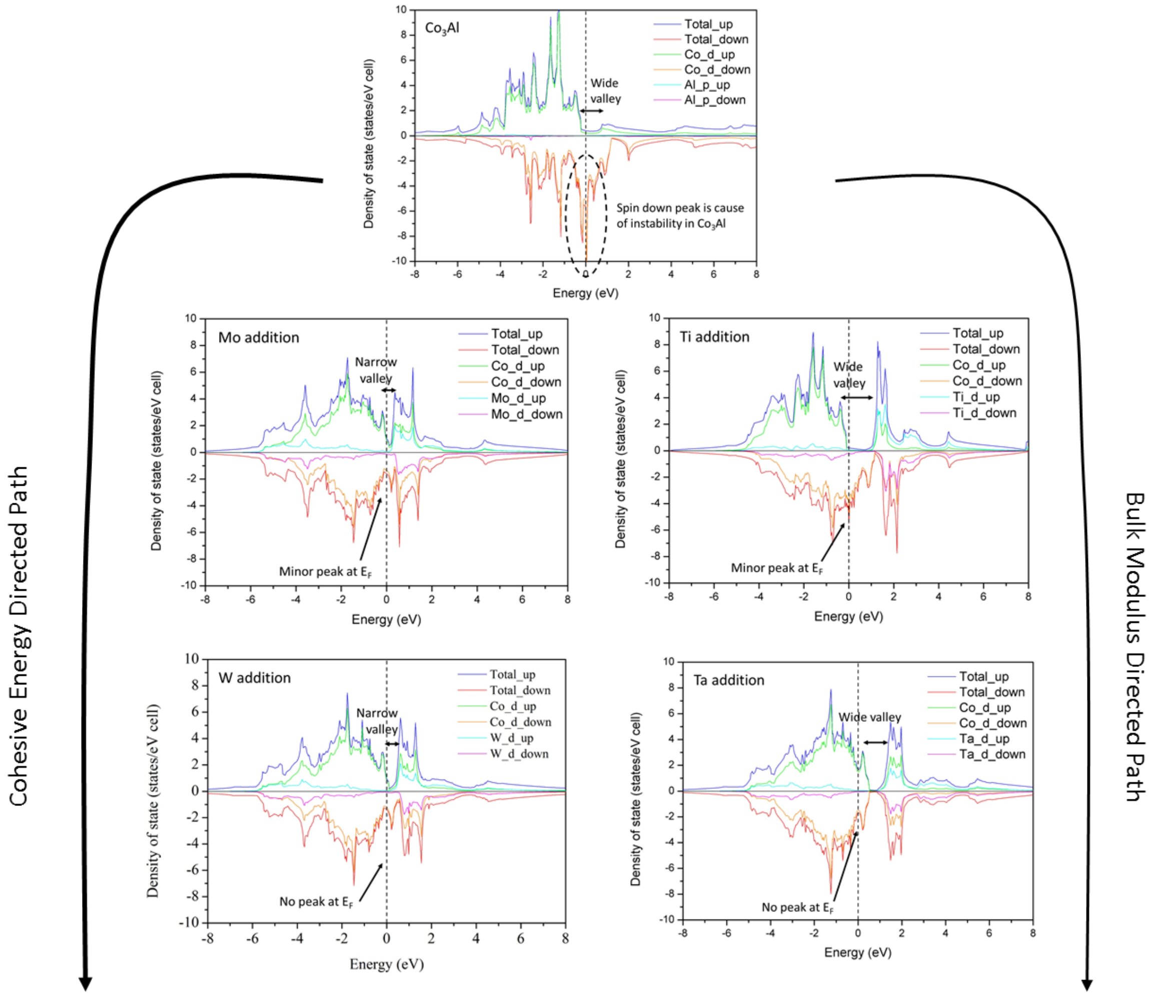

Figure S11. For comparison between selected chemical substitutions and the density of state (DOS), the DOS for different design pathways have been calculated. Co_3Al and Co_3Ti have similar DOS, in terms of shape of total DOS (blue line) at E_F (E_F equal to zero for comparison). The most notable difference between these two DOS is the total DOS down peak at E_F for Co_3Al (shown as region of dashed circle), which explains the instability of Co_3Al in the $L1_2$ structure. This feature is not seen in Co_3Ti which shows some stability at ground state. The DOS of Co_3W and Co_3Mo is also seen to be similar using the same criteria. Further examination of the DOS at E_F shows a valley for each system. However, the valley is much more defined for Co_3Ta and Co_3Ti (bulk modulus pathway), than it is for Co_3W and Co_3Mo (cohesive energy pathway). This explanation contributes to the similarity between Co_3Al and $Co_3(Al,Ti)$ in our graph network, while $Co_3(Al,Mo)$, $Co_3(Al,Ta)$, $Co_3(Al,W)$ are nodes separated, yet more stable, from Co_3Al.

References

[S1] Ashby, M.F., *Proc. R. Soc. Lond. A*, **454** (1998) 1301.
[S2] Benfey, O.T., J. Chem. Educ., **42** (1965) 39.
[S3] Emsley, J., *The Element*. (Oxford University Press) 1998.
[S4] Jensen, W.B., Classification, symmetry and the periodic table, in *Unifying Human Understanding*, ed. I. Hargittai. Paramount Press. 1986.
[S5] Kittel, C., *Introduction to Solid State Physics*. (John Wiley & Sons, Inc) 1996.
[S6] Rabe, K.M., J.C. Phillips, P. Villars, I.D. Brown, *Phys. Rev. B*, **45** (1992) 7650.
[S7] Restrepo, G., H. Mesa, E.J. Llanos, J.L. Villaveces, *J. Chem. Inf. Comput. Sci*., **44** (2004) 68.
[S8] Tiwari, G.P., R.V. Ramanujan, *J. Mater. Sci.*, **36** (2001) 271.
[S9] Von Hippel, A., *The Molecular Designing of Materials and Devices*, M.I.T. Press. (1965)
[S10] W. Hume-Rothery, *Prog. Mater. Sci.* **13** (1968) 229–65.
[S11] P. Villars, *Intermetallic Compounds: Principles and Practice*, J.H. Westbrook and R.L. Fleischer, eds. (New York: Wiley) 1995
[S12] W. Pearson, *Crystal Chemistry and Physics of Metals and Alloys* (New York: Wiley), 1972.
[S13] E. Mooser, W.B. Pearson, *Acta Crystallogr*., **12** (1959) 1015.
[S14] B. Seiser, R. Drautz, D.G. Pettifor, *Acta Mat* **59** (2011) 749-763.
[S15] A.R. Miedema, A.K. Niessen, F.R. de Boer, R. Boom, W.C.M. Matten, *Cohesion in Metals: Transition Metal Alloys*, (North-Holland, Amsterdam), 1989**.**
[S16] G. Kresse and J. Furthmüller, Comput. Mater. Sci. **6**, 15 (1996).
[S17] G. Kresse and J. Furthmüller, Phys. Rev. B **54**, 11169 (1996).
[S18] J. P. Perdew, J. A. Chevary, S. H. Vosko, K. A. Jackson, M. R. Pederson, D. J. Singh, and C. Fiolhais, Phys. Rev. B 46, 6671 (1992).
[S19] P. E. Blöchl, Phys. Rev. B 50, 17953 (1994).
[S20] G. Kresse and D. Joubert, Phys. Rev. B 59, 1758 (1999).
[S21] S. R. Broderick and K. Rajan, *Europhys. Lett.* **95** (2011) 57005.
[S22] S. R. Broderick, H. Aourag, and K. Rajan, *J. Am. Ceram. Soc.* **94** (2011) 2974.
[S23] S.R. Broderick, H. Aourag, K. Rajan, *Stat. Analys. Data Mining* **6** (2009) 353-360
[S24] Wagaman, A., and Levina, E. Discovering sparse covariance structures with the isomap. *Journal of Computational and Graphical Statistics 18*, 551-572 (2009).
[S25] Cukierski, W.J.; Foran, D.J. In *Using betweenness centrality to identify manifold shortcuts*, Data Mining Workshops, 2008. ICDMW'08. IEEE International Conference on, 2008; IEEE: 2008; pp. 949-958.
[S26] M. Balasubramanian, E.L. Schwartz, *Science* **2002**, *295*, 7-7.
[S27] A. Suzuki, T.M. Pollock. *Acta Mat* **56**, 1288-1297 (2008)
